# Supplementary material for: Aberrant computational mechanisms of social learning and decision-making in schizophrenia and borderline personality disorder
Source: PLoS Comput Biol. 2020 Sep 30;16(9):e1008162. doi: 10.1371/journal.pcbi.1008162 (PMC7588082; doi:10.1371/journal.pcbi.1008162)
Supplement: S1 Table — All quantities given as Mean ± SD. (DOCX) [file pcbi.1008162.s001.docx]

**S1 Table. Psychometric data of the participants.** All quantities given as Mean ± SD.

|  | **HC Participants** | **MDD Participants** | **SCZ Participants** | **BPD Participants** | **Significance** |
| --- | --- | --- | --- | --- | --- |
|  |  |  |  |  |  |
| **n** | **31** | **28** | **29** | **28** |  |
| **Gender,**  **(m/f)** | (15/16) | (13/15) | (23/6) | (8/20) | ${{}^{2}(3)}$= 15.25,  *P* = 0.002  (Chi-Square test) |
| **Age,**  **mean (SD)** | 35.65 (12.97) | 38.43 (10.69) | 33.59 (10.01) | 31.32 (7.88) | ${{}^{2}(3)}$= 5.302,  *P* = 0.151  (Kruskal-Wallis’one way ANOVA) |
| **Years of school,**  **mean (SD)** | 12.06 (1.99) | 12.32 (2.21) | 10.87 (3.965) | 10.74 (1.973) | F(3,109)= 2.621,  *P* = 0.054 (ANOVA)^a^ |
| **AQ,**  **mean (SD)** | 16.66 (5.48) | 22.24 (8.23) | 21.61 (6.66) | 24.72 (6.52) | F(3,103)=7.262,  *P* <.001 (ANOVA)^b^ |
| **ACIPS,**  **mean (SD)** | 81.23 (11.75) | 68.57 (16.45) | 74.11 (15.1) | 66 (16.6) | F(3,103)=5.719, *P* <.001  (ANOVA)^b^ |
| **CDSS,**  **mean (SD)** | -^c^ | - | 4.11 (4.00) | - | - |
| **PANSS, Positive,**  **mean (SD)** | - | - | 11.45 (3.43) | - | - |
| **PANSS, Negative,**  **mean (SD)** | - | - | 13.86 (4.21) | - | - |
| **PANSS, General,**  **mean (SD)** | - | - | 26.59 (5.82) | - | - |
| **PANSS, Total, mean (SD)** | - | - | 52.24 (11.25) | - | - |
| **BSL-23 (sum), mean (SD)** | ---^i^ | --- | --- | 45.4 (22.59) ^d^ |  |

^a^ Three missing data points. ^b^ Nine missing data points. ^c^ One hyphen indicates that measure applies only to Participants with SCZ. ^d^ three missing data points.
