# Supplementary material for: Aberrant computational mechanisms of social learning and decision-making in schizophrenia and borderline personality disorder
Source: PLoS Comput Biol. 2020 Sep 30;16(9):e1008162. doi: 10.1371/journal.pcbi.1008162 (PMC7588082; doi:10.1371/journal.pcbi.1008162)
Supplement: S2 Table — All quantities given as Mean ± SD. (DOCX) [file pcbi.1008162.s002.docx]

**S2 Table. Demographic data of the participants.** All quantities given as Mean ± SD.

|  | **HC Participants** | **MDD Participants** | **SCZ Participants** | **BPD Participants** |
| --- | --- | --- | --- | --- |
| **Age at Diagnosis,**  **mean (SD)** | -^a^ | 28.3(11.62)^b^ | 26.25 ± 9.82^b^ | 15.15(5.5)^b^ |
| **Number of Hospitalizations,**  **mean (SD)** | - | 3.14(4.07) | 5.88(5.9)^c^ | 6.14(6) |
| **Duration current Hospitalization (days), mean (SD)** | - | 20.79(16.63) | 65.19(49.74)^d^ | 13.75(10.71) |
| **Relationship Status**  **No. (%)** |  |  |  |  |
| **None** | 12(38.71) | 11(39.29) | 19(65.52) | 14(50) |
| **In a relationship** | 16(51.61) | 4(14.29) | 7(24.14) | 10(35.71) |
| **Married** | 2(6.45) | 11(39.29) | 3(10.35) | 2 (7.14) |
| **Divorced** | 1(3.23) | 0(0) | 0(0) | 2(7.14) |
| **Widowed** | 0(0) | 0(0) | 0(0) | 0(0) |
| **No answer** | 0(0) | 1(3.57) | 0(0) | 0(0) |
| **Employment Status**  **No. (%)** |  |  |  |  |
| **Regularly employed** | 19(61.3) | 13(46.43) | 3(10.35) | 7(25) |
| **Unemployed** | 2(6.5) | 7(25) | 12(41.38) | 14(50) |
| **Unable to work** | 0(0) | 3(10.71) | 4(13.8) | 1(3.6) |
| **Supervised work** | 0(0) | 1(3.571) | 3(10.35) | 0(0) |
| **Retired** | 0(0) | 1(3.571) | 2(6.9) | 3(10.71) |
| **In school** | 10(32.26) | 0(0) | 3(10.35) | 3(10.71) |
| **No answer** | 0(0) | 2(7.24) | 2(6.9) | 0(0) |
| **Immigration Status**  **No. (%)** |  |  |  |  |
| **Native** | 28(90.32) | 18(64.29) | 15(51.72) | 18(64.29) |
| **Migrant** | 3(9.68) | 8(28.571) | 14(48.28) | 9(32.14) |
| **Neuroactive Medications**  **No. (%)^d^** |  |  |  |  |
| **Taking Psychiatric Medications** | 0(0) | 27(96.43) | 26(89.66) | 26(92.86) |
| **Antidepressants only** | 0(0) | 13(46.43) | 0(0) | 10(35.71) |
| **Antipsychotics only** | 0(0) | 0(0) | 17(58.62) | 2(7.14) |
| **Antidepressants and Antipsychotics (Combination)** | 0(0) | 13(46.43) | 9(31.03) | 13(46.43) |
| **Mood Stabilizer** | 0(0) | 6(21.43) | 1(3.45) | 1(3.57) |
| **Sedatives** | 0(0) | 0(0) | 1(3.45) | 2(7.14) |
| **Other** | 0(0) | 3(10.71) | 6(20.69) | 5(17.86) |
| **Chlorpromazine Equivalents (in mg)** | 0(0) | 47.27(90.78) | 432.1(371.8) | 51.26(81.78) |

^a^ One hyphen indicates that the measure only applies to patients. ^b^ One missing data point. ^c^ Four missing data points. ^d^ Thirteen participants with SCZ were not recruited during hospitalization.
