# Supplementary material for: Aberrant computational mechanisms of social learning and decision-making in schizophrenia and borderline personality disorder
Source: PLoS Comput Biol. 2020 Sep 30;16(9):e1008162. doi: 10.1371/journal.pcbi.1008162 (PMC7588082; doi:10.1371/journal.pcbi.1008162)
Supplement: S3 Table — Means and variances of Gaussian priors are given in the space in which the parameter was estimated (native, log, or logit). (DOCX) [file pcbi.1008162.s003.docx]

**S3 Table**. **Prior configurations of perceptual and response model parameters.** Means and variances of Gaussian priors are given in the space in which the parameter was estimated (native, log, or logit).

| **HGF** | | **Level 1** | | **Level 2** | | **Level 3** | |
| --- | --- | --- | --- | --- | --- | --- | --- |
| **Parameter** | **Estimation Space** | **Prior Mean** | **Prior Variance** | **Prior Mean** | **Prior Variance** | **Prior Mean** | **Prior Variance** |
| $\boldsymbol{\mu}^{\boldsymbol{(k=0)}}$ | **native** | - | - | 0 | 0 | 1 | 0 |
| $\boldsymbol{\sigma}^{\boldsymbol{(k=0)}}$ | **log** | - | - | log (0.4) | 1 | log (0.1) | 1 |
| $\boldsymbol{\varphi}$ | **logit** | - | - | logit (0) | 0 | logit (0.1) | 2 |
| $\boldsymbol{m}{}$ | **native** | - | - | 0 | 0 | 1 | 0 |
| $\boldsymbol{\kappa}$ | **log** | log(1) | 0 | log (1) | 0 | - | - |
| $\boldsymbol{\omega}$ | **native** | - | - | -4 | 4 | -6 | 4 |
| **ST-K1** | **Estimation Space** | **Prior Mean** | **Prior Variance** |  |  |  |  |
| $\boldsymbol{\mu}$ | **log** | log (1) | 1 |  |  |  |  |
| $\hat{\boldsymbol{r}}$ | **log** | log (1) | 0 |  |  |  |  |
| $\hat{\boldsymbol{v}}$ | **logit** | logit (0.5) | 16 |  |  |  |  |
| $\boldsymbol{h}$ | **logit** | logit (0.005) | 16 |  |  |  |  |
| **RW** | **Estimation Space** | Prior Mean | Prior Variance |  |  |  |  |
| $\boldsymbol{\nu}^{\boldsymbol{(k=0)}}$ | **logit** | logit (0.5) | 1 |  |  |  |  |
| $\boldsymbol{\alpha}$ | **logit** | logit (0.5) | 1 |  |  |  |  |
| **Response Model** | **Estimation Space** | **Prior Mean** | **Prior Variance** |  |  |  |  |
| $\boldsymbol{\zeta}$ | **log** | log (1) | 16 |  |  |  |  |
| $\boldsymbol{\beta}$ | **log** | log (16) | 16 |  |  |  |  |
|  | **logit** | logit (0.5) | 2 |  |  |  |  |
