# Supplementary material for: Aberrant computational mechanisms of social learning and decision-making in schizophrenia and borderline personality disorder
Source: PLoS Comput Biol. 2020 Sep 30;16(9):e1008162. doi: 10.1371/journal.pcbi.1008162 (PMC7588082; doi:10.1371/journal.pcbi.1008162)
Supplement: S4 Table — (DOCX) [file pcbi.1008162.s004.docx]

**S4 Table**. **Within-subjects model comparison.**

|  | **HGF 1** | **HGF 2** | **HGF 3** | **HGF 4** | **ST-K** | **RW** | **WSLS** | **Random** |
| --- | --- | --- | --- | --- | --- | --- | --- | --- |
| **HC** | 8 | 5 | 2 | 0 | 8 | 5 | 2 | 1 |
| **MDD** | 10 | 3 | 0 | 0 | 4 | 2 | 6 | 3 |
| **SCZ** | 14 | 0 | 1 | 0 | 2 | 3 | 2 | 7 |
| **BPD** | 15 | 0 | 1 | 0 | 2 | 4 | 1 | 5 |
| **All** | 47 | 8 | 4 | 0 | 16 | 14 | 11 | 16 |
