# Supplementary material for: Aberrant computational mechanisms of social learning and decision-making in schizophrenia and borderline personality disorder
Source: PLoS Comput Biol. 2020 Sep 30;16(9):e1008162. doi: 10.1371/journal.pcbi.1008162 (PMC7588082; doi:10.1371/journal.pcbi.1008162)
Supplement: S5 Table — (DOCX) [file pcbi.1008162.s005.docx]

**S5 Table. Mean posterior estimates of learning model and decision model parameters estimated from winning model.**

| **Parameter** |  | $\boldsymbol{\omega}_{\boldsymbol{2}\boldsymbol{card}}$ | $\boldsymbol{\omega}_{\boldsymbol{2}\boldsymbol{gaze}}$ | $\boldsymbol{\omega}_{\boldsymbol{3}\boldsymbol{card}}$ | $\boldsymbol{\omega}_{\boldsymbol{3}\boldsymbol{gaze}}$ | $\mathbf{log(}\boldsymbol{\zeta)}$ | $\mathbf{log}\boldsymbol{(\beta)}$ | $\mathbf{logit}\boldsymbol{(\eta)}$ |
| --- | --- | --- | --- | --- | --- | --- | --- | --- |
| **mean (SD)** | HC | -2.810 (1.838) | -3.129 (1.663) | -5.943 (0.253) | -6.043 (0.508) | -1.155 (2.043) | 2.205 (0.796) | -0.201 (0.917) |
|  | MDD | -2.875 (1.919) | -3.773 (1.694) | -6.032 (0.281) | -6.049 (0.191) | -0.9276 (1.706) | 1.634 (0.897) | -0.054 (0.395) |
|  | SCZ | -3.3422 (1.172) | -3.962 (2.155) | -5.956 (0.246) | -5.927 (0.221) | 0.348 (2.365) | 1.297 (1.184) | -0.169 (0.545) |
|  | BPD | -4.000 (1.525) | -4.941 (2.688) | -5.992 (0.070) | -6.041 (0.141) | 0.750 (2.313) | 1.690 (1.049) | -0.078 (0.697) |
