# Supplementary material for: Aberrant computational mechanisms of social learning and decision-making in schizophrenia and borderline personality disorder
Source: PLoS Comput Biol. 2020 Sep 30;16(9):e1008162. doi: 10.1371/journal.pcbi.1008162 (PMC7588082; doi:10.1371/journal.pcbi.1008162)
Supplement: S6 Table — The table shows the results for the real and simulated behavior. (DOCX) [file pcbi.1008162.s006.docx]

**S6 Table. Statistics for mixed ANOVA with response accuracy (% High probability choices) from real and simulated data for stable and volatile phases (Factor Phase) of social and non-social cue (Factor Cue Type) for all groups (Factor Group) and schedules (Factor Schedule). The table shows the results for the real and simulated behavior.**

| **ANOVA - Real Behaviour** |  | | | | |
| --- | --- | --- | --- | --- | --- |
| **Within Subjects Effects** |  |  |  |  |  |
|  | **df** | **Mean Square** | **F** | **p** | **η²** |
| Cue Type | 1 | 165.428 | 2.577 | 0.111 | 0.019 |
| Cue Type x Group | 3 | 309.462 | 4.820 | 0.003 | 0.108 |
| Cue Type x Schedule | 1 | 439.580 | 6.847 | 0.010 | 0.051 |
| Cue Type x Group x Schedule | 3 | 39.776 | 0.620 | 0.604 | 0.014 |
| Residual | 108 | 64.200 |  |  |  |
| Phase | 1 | 32.911 | 0.266 | 0.607 | 0.002 |
| Phase x Group | 3 | 227.070 | 1.834 | 0.145 | 0.047 |
| Phase x Schedule | 1 | 180.771 | 1.460 | 0.230 | 0.012 |
| Phase x Group x Schedule | 3 | 74.238 | 0.599 | 0.617 | 0.015 |
| Residual | 108 | 123.844 |  |  |  |
| Cue Type x Phase | 1 | 6.238 | 0.101 | 0.751 | 0.001 |
| Cue Type x Phase x Group | 3 | 145.200 | 2.359 | 0.076 | 0.049 |
| Cue Type x Phase x Schedule | 1 | 1.539.536 | 25.016 | < .001 | 0.171 |
| Cue Type x Phase x Group x Schedule | 3 | 117.458 | 1.909 | 0.133 | 0.039 |
| Residual | 108 | 61.541 |  |  |  |
| **Between Subjects Effects** |  | | | | |
| **Cases** | **df** | **Mean Square** | **F** | **p** | **η²** |
| Group | 3 | 1165.8 | 4.945 | 0.003 | 0.116 |
| Schedule | 1 | 433.7 | 1.840 | 0.178 | 0.014 |
| Group x Schedule | 3 | 210.5 | 0.893 | 0.447 | 0.021 |
| Residual | 108 | 235.7 |  |  |  |
| **Post Hoc Comparisons - Group** |  | | | | |
|  | **Mean Difference** | **SE** | **t** | **p _bonf_** | **Cohen's d** |
| HC vs. MDD | 3.792 | 2.005 | 1.892 | 0.367 | 0.176 |
| HC vs. SCZ | 6.694 | 1.984 | 3.373 | 0.006 | 0.313 |
| HC vs. BPD | 6.470 | 2.005 | 3.227 | 0.010 | 0.300 |
| MDD vs. SCZ | 2.902 | 2.037 | 1.425 | 0.943 | 0.132 |
| MDD vs. BPD | 2.678 | 2.057 | 1.302 | 1.000 | 0.121 |
| SCZ vs. BPD | -0.224 | 2.037 | -0.110 | 1.000 | -0.010 |
| **ANOVA - Simulated Behaviour** |  | | | | |
| **Within Subjects Effects** |  |  |  |  |  |
|  | **df** | **Mean Square** | **F** | **p** | **η²** |
| Cue Type | 1 | 85.120 | 4.066 | 0.046 | 0.027 |
| Cue Type x Group | 3 | 229.874 | 10.981 | < .001 | 0.219 |
| Cue Type x Schedule | 1 | 90.595 | 4.327 | 0.040 | 0.029 |
| Cue Type x Group x Schedule | 3 | 9.487 | 0.453 | 0.716 | 0.009 |
| Residual | 108 | 20.935 |  |  |  |
| Phase | 1 | 48.598 | 2.991 | 0.087 | 0.022 |
| Phase x Group | 3 | 97.866 | 6.023 | < .001 | 0.131 |
| Phase x Schedule | 1 | 137.825 | 8.482 | 0.004 | 0.061 |
| Phase x Group x Schedule | 3 | 2.774 | 0.171 | 0.916 | 0.004 |
| Residual | 108 | 16.250 |  |  |  |
| Cue Type x Phase | 1 | 202.349 | 4.629 | 0.034 | 0.037 |
| Cue Type x Phase x Group | 3 | 123.402 | 2.823 | 0.042 | 0.068 |
| Cue Type x Phase x Schedule | 1 | 103.654 | 2.371 | 0.127 | 0.019 |
| Cue Type x Phase x Group x Schedule | 3 | 8.874 | 0.203 | 0.894 | 0.005 |
| Residual | 108 | 43.717 |  |  |  |
| **Between Subjects Effects** |  | | | | |
| **Cases** | **df** | **Mean Square** | **F** | **p** | **η²** |
| Group | 3 | 587.10 | 6.755 | < .001 | 0.149 |
| Schedule | 1 | 38.04 | 0.438 | 0.510 | 0.003 |
| Group x Schedule | 3 | 218.54 | 2.514 | 0.062 | 0.055 |
| Residual | 108 |  |  |  |  |
| **Post Hoc Comparisons - Group** |  | | | | |
|  | **Mean Difference** | **SE** | **t** | **p _bonf_** | **Cohen's d** |
| HC vs. MDD | 2.939 | 1.217 | 2.415 | 0.105 | 0.224 |
| HC vs. SCZ | 4.868 | 1.205 | 4.040 | < .001 | 0.375 |
| HC vs. BPD | 4.473 | 1.217 | 3.675 | 0.002 | 0.341 |
| MDD vs. SCZ | 1.929 | 1.237 | 1.559 | 0.731 | 0.145 |
| MDD vs. BPD | 1.534 | 1.249 | 1.228 | 1.000 | 0.114 |
| SCZ vs. BPD | -0.395 | 1.237 | -0.319 | 1.000 | -0.030 |
