# Supplementary material for: Aberrant computational mechanisms of social learning and decision-making in schizophrenia and borderline personality disorder
Source: PLoS Comput Biol. 2020 Sep 30;16(9):e1008162. doi: 10.1371/journal.pcbi.1008162 (PMC7588082; doi:10.1371/journal.pcbi.1008162)
Supplement: S7 Table — The table shows the results for the full and reduced sample. (DOCX) [file pcbi.1008162.s007.docx]

**S7 Table. Statistics for mixed ANOVA with averaged *q*(**$\boldsymbol{\psi}_{\boldsymbol{2}}\boldsymbol{)}$ **during stable and volatile phases (Factor Phase) of social and non-social cue (Factor Cue Type) for all groups (Factor Group) and schedules (Factor Schedule). The table shows the results for the full and reduced sample.**

| **ANOVA - Full Sample** |  |  |  |  |  |
| --- | --- | --- | --- | --- | --- |
| **Within Subjects Effects** |  | | | | |
|  | **df** | **Mean Square** | **F** | **p** | **η²** |
| Cue Type | 1 | 0.029 | 1.651 | 0.202 | 0.014 |
| Cue Type x Group | 3 | 0.018 | 1.038 | 0.379 | 0.027 |
| Cue Type x Schedule | 1 | 0.031 | 1.788 | 0.184 | 0.016 |
| Cue Type x Group x Schedule | 3 | 0.004 | 0.204 | 0.894 | 0.005 |
| Residual | 108 | 0.017 |  |  |  |
| Phase | 1 | 0.006 | 24.868 | < .001 | 0.182 |
| Phase x Group | 3 | 1.951e -4 | 0.828 | 0.481 | 0.018 |
| Phase x Schedule | 1 | 1.990e -4 | 0.844 | 0.360 | 0.006 |
| Phase x Group x Schedule | 3 | 6.224e -5 | 0.264 | 0.851 | 0.006 |
| Residual | 108 | 2.357e -4 |  |  |  |
| Cue Type x Phase | 1 | 3.563e -5 | 0.131 | 0.718 | 0.001 |
| Cue Type x Phase x Group | 3 | 2.549e -4 | 0.940 | 0.424 | 0.025 |
| Cue Type x Phase x Schedule | 1 | 1.798e -4 | 0.663 | 0.417 | 0.006 |
| Cue Type x Phase x Group x Schedule | 3 | 1.716e -4 | 0.633 | 0.595 | 0.017 |
| Residual | 108 | 2.710e -4 |  |  |  |
| **Between Subjects Effects** |  |  |  |  |  |
| **Cases** | **df** | **Mean Square** | **F** | **p** | **η²** |
| Group | 3 | 0.088 | 3.557 | 0.017 | 0.088 |
| Schedule | 1 | 0.022 | 0.901 | 0.345 | 0.007 |
| Group x Schedule | 3 | 0.011 | 0.441 | 0.724 | 0.011 |
| Residual | 108 | 0.025 |  |  |  |
| **Post Hoc Comparisons - Group** |  |  |  |  |  |
|  | **Mean Difference** | **SE** | **t** | **p _bonf_** | **Cohen's d** |
| HC vs. MDD | 0.018 | 0.021 | 0.877 | 1.000 | 0.081 |
| HC vs. SCZ | 0.039 | 0.020 | 1.938 | 0.332 | 0.180 |
| HC vs. BPD | 0.064 | 0.021 | 3.101 | 0.015 | 0.288 |
| MDD vs. SCZ | 0.021 | 0.021 | 1.024 | 1.000 | 0.095 |
| MDD vs. BPD | 0.046 | 0.021 | 2.167 | 0.195 | 0.201 |
| SCZ vs. BPD | 0.024 | 0.021 | 1.164 | 1.000 | 0.108 |
| **Post Hoc Comparisons - Phase** |  | | | | |
| Stable vs vol | -0.007 | 0.001 | -5.147 | < .001 | -0.478 |
| **ANOVA - Reduced Sample** |  | | | | |
| **Within Subjects Effects** |  |  |  |  |  |
|  | **df** | **Mean Square** | **F** | **p** | **η²** |
| Cue Type | 1 | 0.023 | 1.202 | 0.276 | 0.012 |
| Cue Type x Group | 3 | 0.024 | 1.264 | 0.291 | 0.038 |
| Cue Type x Schedule | 1 | 0.033 | 1.741 | 0.190 | 0.017 |
| Cue Type x Group x Schedule | 3 | 0.008 | 0.446 | 0.721 | 0.013 |
| Residual | 92 | 0.019 |  |  |  |
| Phase | 1 | 0.005 | 18.690 | < .001 | 0.162 |
| Phase x Group | 3 | 2.760e -4 | 1.018 | 0.388 | 0.027 |
| Phase x Schedule | 1 | 1.742e -4 | 0.643 | 0.425 | 0.006 |
| Phase x Group x Schedule | 3 | 6.035e -5 | 0.223 | 0.880 | 0.006 |
| Residual | 92 | 2.710e -4 |  |  |  |
| Cue Type x Phase | 1 | 2.838e -5 | 0.091 | 0.763 | 0.001 |
| Cue Type x Phase x Group | 3 | 2.662e -4 | 0.858 | 0.466 | 0.027 |
| Cue Type x Phase x Schedule | 1 | 8.859e -5 | 0.286 | 0.594 | 0.003 |
| Cue Type x Phase x Group x Schedule | 3 | 1.145e -4 | 0.369 | 0.776 | 0.012 |
| Residual | 92 | 3.103e -4 |  |  |  |
| **Between Subjects Effects** |  | | | |  |
| **Cases** | **df** | **Mean Square** | **F** | **p** | **η²** |
| Group | 3 | 0.076 | 2.770 | 0.046 | 0.081 |
| Schedule | 1 | 0.038 | 1.388 | 0.242 | 0.014 |
| Group x Schedule | 3 | 0.008 | 0.308 | 0.819 | 0.009 |
| Residual | 92 | 0.028 |  |  |  |
| **Post Hoc Comparisons - Group** |  | | | |  |
|  | **Mean Difference** | **SE** | **t** | **p _bonf_** | **Cohen's d** |
| HC vs. MDD | 0.012 | 0.022 | 0.513 | 1.000 | 0.051 |
| HC vs. SCZ | 0.029 | 0.023 | 1.256 | 1.000 | 0.126 |
| HC vs. BPD | 0.063 | 0.023 | 2.751 | 0.043 | 0.275 |
| MDD vs. SCZ | 0.018 | 0.024 | 0.730 | 1.000 | 0.073 |
| MDD vs. BPD | 0.052 | 0.024 | 2.157 | 0.202 | 0.216 |
| SCZ vs. BPD | 0.034 | 0.025 | 1.375 | 1.000 | 0.137 |
| **Post Hoc Comparisons - Phase** |  | | | | |
| Stable vs vol | -0.007 | 0.002 | -4.398 | < .001 | -0.440 |
