# Supplementary material for: Aberrant computational mechanisms of social learning and decision-making in schizophrenia and borderline personality disorder
Source: PLoS Comput Biol. 2020 Sep 30;16(9):e1008162. doi: 10.1371/journal.pcbi.1008162 (PMC7588082; doi:10.1371/journal.pcbi.1008162)
Supplement: S8 Table — The table shows the results for the full and reduced sample. (DOCX) [file pcbi.1008162.s008.docx]

**S8 Table. Statistics for mixed ANOVA with averaged** $\boldsymbol{\psi}_{\boldsymbol{3}}$ **during stable and volatile phases (Factor Phase) of social and non-social cue (Factor Cue Type) for all groups (Factor Group) and schedules (Factor Schedule). The table shows the results for the full and reduced sample.**

| **ANOVA - Full Sample** |  |  |  |  |  |
| --- | --- | --- | --- | --- | --- |
| **Within Subjects Effects** |  | | | |  |
|  | **df** | **Mean Square** | **F** | **p** | **η²** |
| Cue Type | 1 | 0.563 | 0.954 | 0.331 | 0.008 |
| Cue Type x Group | 3 | 0.726 | 1.231 | 0.302 | 0.032 |
| Cue Type x Schedule | 1 | 0.538 | 0.912 | 0.342 | 0.008 |
| Cue Type x Group x Schedule | 3 | 0.155 | 0.262 | 0.853 | 0.007 |
| Residual | 108 | 0.590 |  |  |  |
| Phase | 1 | 12.670 | 116.206 | < .001 | 0.462 |
| Phase x Group | 3 | 0.650 | 5.962 | < .001 | 0.071 |
| Phase x Schedule | 1 | 0.362 | 3.320 | 0.071 | 0.013 |
| Phase x Group x Schedule | 3 | 0.213 | 1.957 | 0.125 | 0.023 |
| Residual | 108 | 0.109 |  |  |  |
| Cue Type x Phase | 1 | 0.258 | 2.862 | 0.094 | 0.024 |
| Cue Type x Phase x Group | 3 | 0.236 | 2.620 | 0.055 | 0.065 |
| Cue Type x Phase x Schedule | 1 | 0.152 | 1.692 | 0.196 | 0.014 |
| Cue Type x Phase x Group x Schedule | 3 | 0.028 | 0.309 | 0.819 | 0.008 |
| Residual | 108 | 0.090 |  |  |  |
| **Between Subjects Effects** |  |  |  |  |  |
| **Cases** | **df** | **Mean Square** | **F** | **p** | **η²** |
| Group | 3 | 4.424 | 6.530 | < .001 | 0.141 |
| Schedule | 1 | 3.393 | 5.008 | 0.027 | 0.036 |
| Group x Schedule | 3 | 1.400 | 2.067 | 0.109 | 0.045 |
| Residual | 108 | 0.678 |  |  |  |
| **Post Hoc Comparisons - Group** |  |  |  |  |  |
|  | **Mean Difference** | **SE** | **t** | **p _bonf_** | **Cohen's d** |
| HC vs. MDD | -0.099 | 0.107 | -0.921 | 1.000 | -0.086 |
| HC vs. SCZ | -0.118 | 0.106 | -1.110 | 1.000 | -0.103 |
| HC vs. BPD | -0.452 | 0.107 | -4.204 | < .001 | -0.390 |
| MDD vs. SCZ | -0.019 | 0.109 | -0.175 | 1.000 | -0.016 |
| MDD vs. BPD | -0.353 | 0.110 | -3.199 | 0.011 | -0.297 |
| SCZ vs. BPD | -0.334 | 0.109 | -3.055 | 0.017 | -0.284 |
| **Post Hoc Comparisons - Phase** |  |  |  |  |  |
| Stable vs vol | -0.324 | 0.033 | -9.784 | < .001 | -0.908 |
| **Post Hoc Comparisons - Schedule** |  |  |  |  |  |
| Incongruent first vs. Congruent first | 0.171 | 0.077 | 2.238 | 0.027 | 0.208 |
| **ANOVA - Reduced Sample** |  | | | | |
| **Within Subjects Effects** |  |  |  |  |  |
|  | **df** | **Mean Square** | **F** | **p** | **η²** |
| Cue Type | 1 | 1.224 | 2.012 | 0.159 | 0.020 |
| Cue Type x Group | 3 | 0.676 | 1.112 | 0.348 | 0.033 |
| Cue Type x Schedule | 1 | 0.405 | 0.667 | 0.416 | 0.007 |
| Cue Type x Group x Schedule | 3 | 0.337 | 0.555 | 0.646 | 0.017 |
| Residual | 92 | 0.608 |  |  |  |
| Phase | 1 | 11.424 | 108.867 | < .001 | 0.460 |
| Phase x Group | 3 | 0.943 | 8.991 | < .001 | 0.114 |
| Phase x Schedule | 1 | 0.253 | 2.406 | 0.124 | 0.010 |
| Phase x Group x Schedule | 3 | 0.223 | 2.124 | 0.102 | 0.027 |
| Residual | 92 | 0.105 |  |  |  |
| Cue Type x Phase | 1 | 0.499 | 6.068 | 0.016 | 0.055 |
| Cue Type x Phase x Group | 3 | 0.220 | 2.677 | 0.052 | 0.073 |
| Cue Type x Phase x Schedule | 1 | 0.108 | 1.309 | 0.256 | 0.012 |
| Cue Type x Phase x Group x Schedule | 3 | 0.067 | 0.818 | 0.487 | 0.022 |
| Residual | 92 | 0.082 |  |  |  |
| **Between Subjects Effects** |  |  |  |  |  |
| **Cases** | **df** | **Mean Square** | **F** | **p** | **η²** |
| Group | 3 | 5.946 | 8.481 | < .001 | 0.198 |
| Schedule | 1 | 3.033 | 4.326 | 0.040 | 0.034 |
| Group x Schedule | 3 | 1.623 | 2.315 | 0.081 | 0.054 |
| Residual | 92 | 0.701 |  |  |  |
| **Post Hoc Comparisons - Group** |  |  |  |  |  |
|  | **Mean Difference** | **SE** | **t** | **p _bonf_** | **Cohen's d** |
| HC vs. MDD | -0.113 | 0.113 | -0.999 | 1.000 | -0.100 |
| HC vs. SCZ | -0.150 | 0.118 | -1.276 | 1.000 | -0.128 |
| HC vs. BPD | -0.563 | 0.116 | -4.850 | < .001 | -0.485 |
| MDD vs. SCZ | -0.037 | 0.122 | -0.299 | 1.000 | -0.030 |
| MDD vs. BPD | -0.450 | 0.121 | -3.714 | 0.002 | -0.371 |
| SCZ vs. BPD | -0.413 | 0.125 | -3.307 | 0.008 | -0.331 |
| **Post Hoc Comparisons - Phase** |  |  |  |  |  |
| Stable vs vol | -0.327 | 0.037 | -8.926 | < .001 | -0.893 |
| **Post Hoc Comparisons - Schedule** |  | | | | |
| Incongruent first vs. Congruent first | 0.175 | 0.084 | 2.080 | 0.040 | 0.208 |
