# Supplementary material for: Aberrant computational mechanisms of social learning and decision-making in schizophrenia and borderline personality disorder
Source: PLoS Comput Biol. 2020 Sep 30;16(9):e1008162. doi: 10.1371/journal.pcbi.1008162 (PMC7588082; doi:10.1371/journal.pcbi.1008162)
Supplement: S9 Table — (DOCX) [file pcbi.1008162.s009.docx]

**S9 Table. Statistics for ANCOVA with** $\boldsymbol{\zeta}$**and Chlorpromazine Equivalence Units and Years of School as covariate for full and reduced sample.**

| **ANCOVA - zeta** |  | | | | |
| --- | --- | --- | --- | --- | --- |
| **Full Sample** |  | | | | |
| **Cases** | **df** | **Mean Square** | **F** | **p** | **η²** |
| Group | 3 | 20.228 | 4.707 | 0.004 | 0.111 |
| Schedule | 1 | 31.606 | 7.354 | 0.008 | 0.058 |
| Schuljahre | 1 | 0.126 | 0.029 | 0.864 | 0.000 |
| Group x Schedule | 3 | 3.219 | 0.749 | 0.525 | 0.018 |
| Chlorpromazine | 1 | 1.160 | 0.270 | 0.605 | 0.002 |
| Residual | 103 | 4.298 |  |  |  |
| **Post Hoc Comparisons - Group** |  | | | | |
|  | **Mean Difference** | **SE** | **t** | **p _bonf_** | **Cohen's d** |
| HC vs. MDD | -0.198 | 0.544 | -0.364 | 1.000 | -0.105 |
| HC vs. SCZ | -1.739 | 0.712 | -2.444 | 0.097 | -0.804 |
| HC vs. BPD | -1.785 | 0.558 | -3.196 | 0.011 | -0.816 |
| MDD vs. SCZ | -1.542 | 0.697 | -2.212 | 0.175 | -0.765 |
| MDD vs. BPD | -1.587 | 0.572 | -2.774 | 0.039 | -0.776 |
| SCZ vs. BPD | -0.045 | 0.684 | -0.066 | 1.000 | -0.019 |
| **Post Hoc Comparisons - Schedule** |  |  |  |  |  |
| Incongruent first vs. Congruent first | -1.063 | 0.392 | -2.712 | 0.008 | -0.485 |
| **Reduced Sample** |  | | | | |
| **Cases** | **df** | **Mean Square** | **F** | **p** | **η²** |
| Group | 3 | 24.643 | 5.722 | 0.001 | 0.150 |
| Schedule | 1 | 24.498 | 5.689 | 0.019 | 0.050 |
| Schuljahre | 1 | 2.079 | 0.483 | 0.489 | 0.004 |
| Group x Schedule | 3 | 3.057 | 0.710 | 0.549 | 0.019 |
| Chlorpromazine | 1 | 4.208 | 0.977 | 0.326 | 0.009 |
| Residual | 88 | 4.306 |  |  |  |
| **Post Hoc Comparisons - Group** |  | | | | |
|  | **Mean Difference** | **SE** | **t** | **p _bonf_** | **Cohen's d** |
| HC vs. MDD | -0.179 | 0.566 | -0.316 | 1.000 | -0.093 |
| HC vs. SCZ | -2.157 | 0.781 | -2.763 | 0.042 | -0.982 |
| HC vs. BPD | -2.007 | 0.588 | -3.415 | 0.006 | -0.922 |
| MDD vs. SCZ | -1.979 | 0.767 | -2.580 | 0.069 | -0.977 |
| MDD vs. BPD | -1.828 | 0.614 | -2.975 | 0.023 | -0.909 |
| SCZ vs. BPD | 0.151 | 0.772 | 0.195 | 1.000 | 0.064 |
| **Post Hoc Comparisons - Schedule** |  |  |  |  |  |
| Incongruent first vs. Congruent first | -1.024 | 0.429 | -2.385 | 0.019 | -0.456 |
