# Supplementary figures and images for: Aberrant computational mechanisms of social learning and decision-making in schizophrenia and borderline personality disorder
Source: PLoS Comput Biol. 2020 Sep 30;16(9):e1008162. doi: 10.1371/journal.pcbi.1008162 (PMC7588082; doi:10.1371/journal.pcbi.1008162)

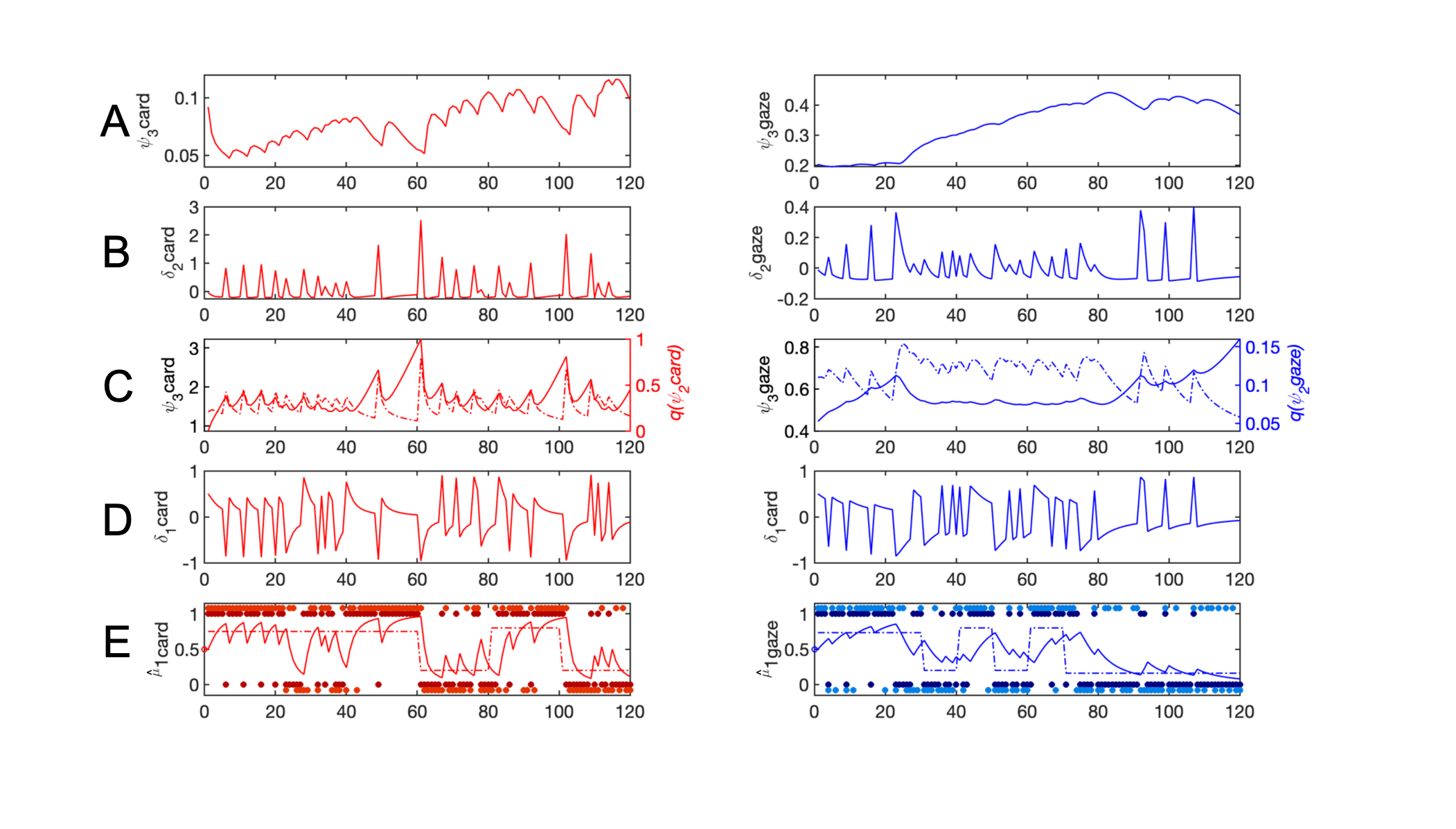

Supplement: S1 Fig — A, Precisions ψ3card (red) and ψ3gaze (blue) that modulate the weight on B, prediction errors δ2card (red) and δ2gaze (blue). C, Precision weights ψ2card in red trajectory and q(ψ2card) in red dotted trajectory. Precision weights ψ2gaze in blue trajectory and q(ψ2gaze) in blue dotted trajectory. Precision weights modulate weight on D) prediction error δ1card (red) and δ1gaze (blue) signals. E, Dark red dots mark the input structure of the non-social information (blue correct = 1; green correct = 0) and the dotted red line represents the ground truth of this input structure. Light red dots mark the choices (blue card = 1; green card = 0). The red trajectory is the participant specific belief trajectory about the blue card to be correct that was estimated on the basis of the choices. E, The same logic applies to the social input and response structure in blue. The posterior parameter estimates for this particular participant were ω2card = -1.460, ω2gaze = -3.576, ω3card = -6.021, ω2gaze = -6.074, log(ζ) = -2.0243, log(β) = 2.207, logit(η) = 0.211. (TIF) [file pcbi.1008162.s010.tif]

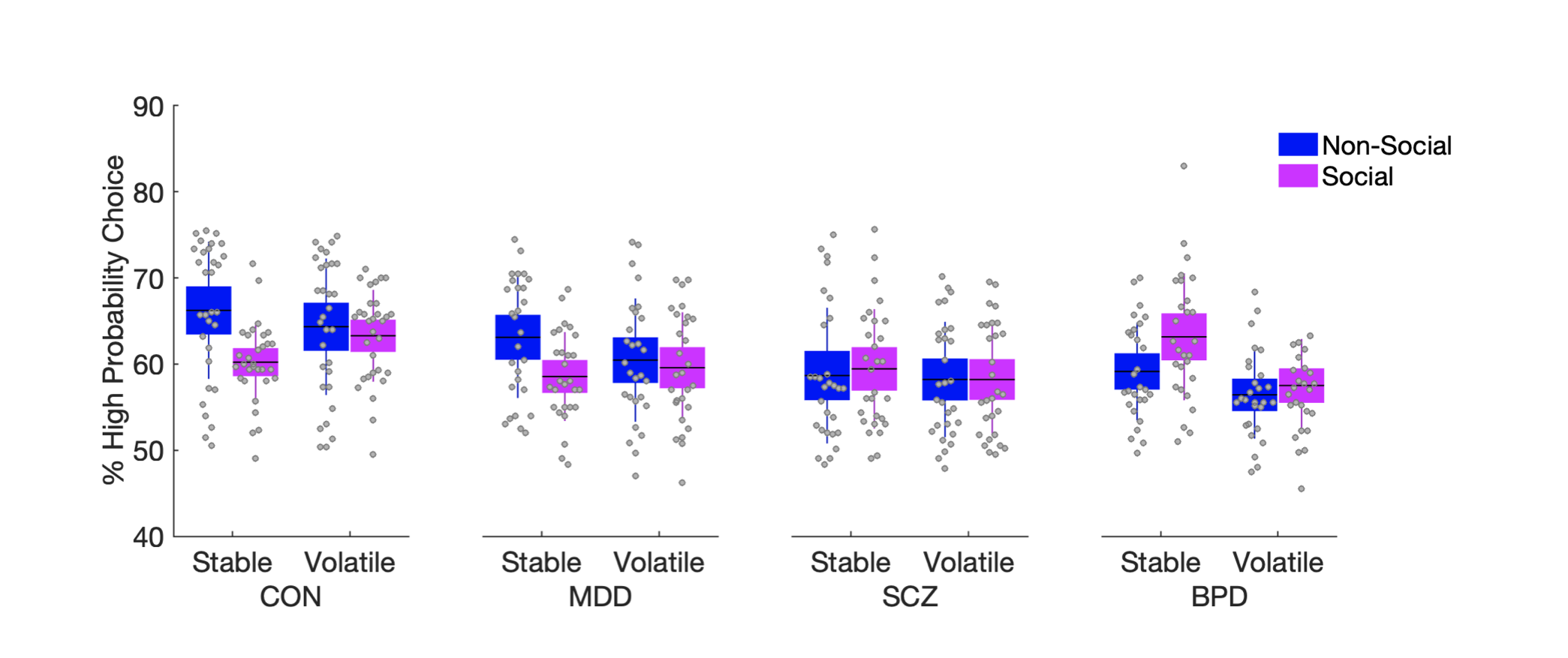

Supplement: S2 Fig — Simulated Behavior from the posterior estimates of all participants revealed the same effects as real behavior. Boxes mark 95% confidence intervals and vertical lines standard deviations. (TIF) [file pcbi.1008162.s011.tif]

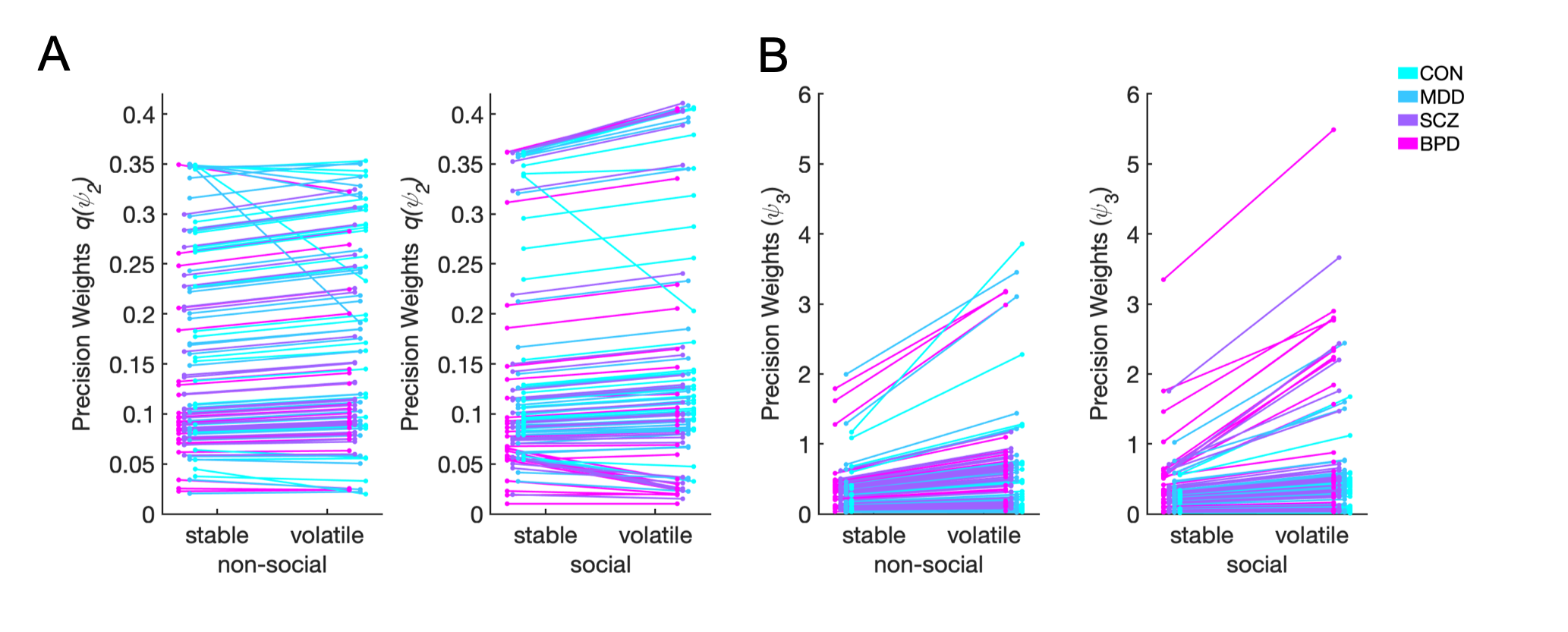

Supplement: S3 Fig — A, precision weights q(ψ2). B, precision weights ψ3. Overall, q(ψ2) and ψ3 increase when transitioning from stable to volatile phase. (TIF) [file pcbi.1008162.s012.tif]

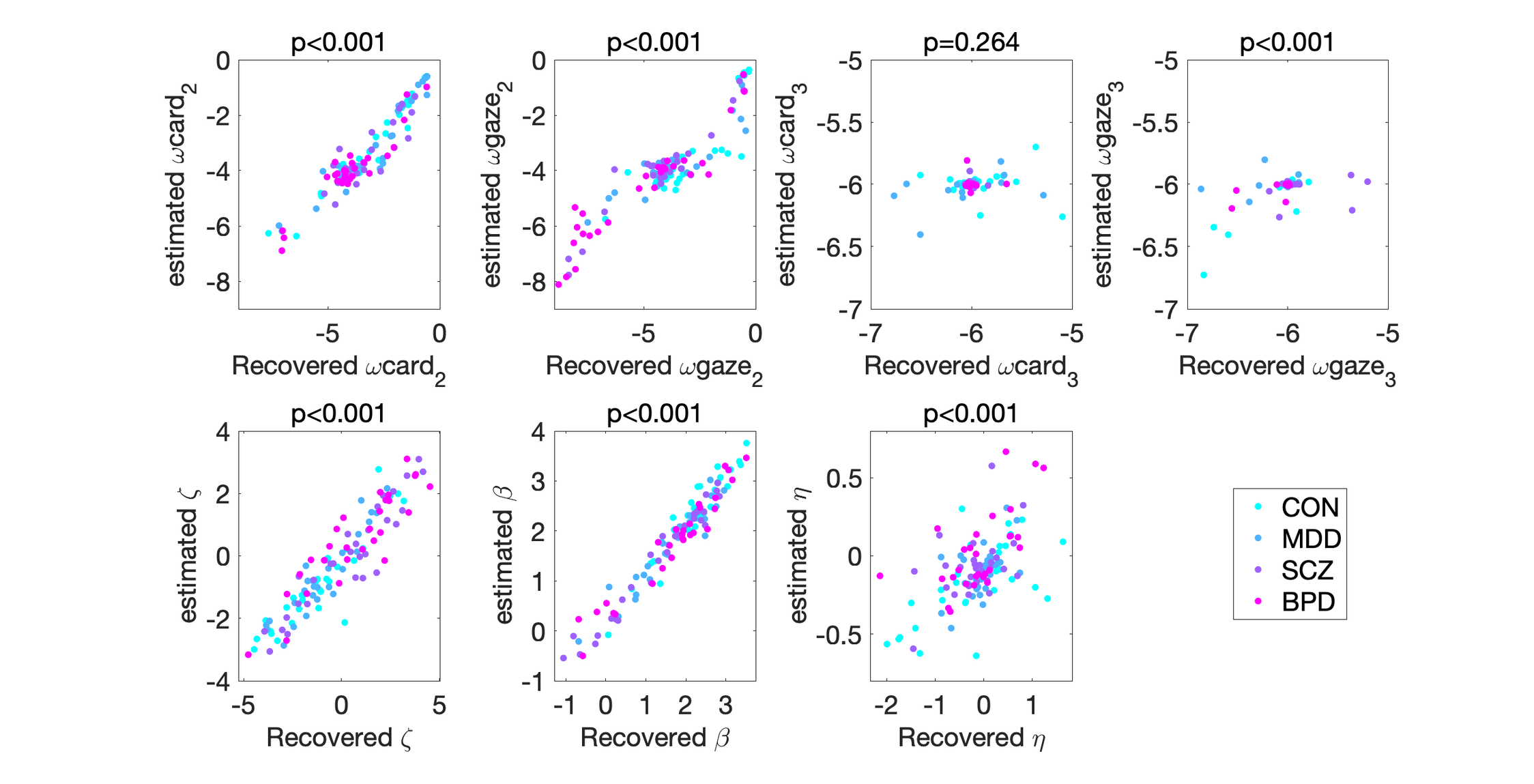

Supplement: S4 Fig — We simulated behavioral responses based on the posterior estimates of all participants 10 times, resulting in 1160 simulations. For each subject, we calculated the average posteriors estimated from the simulated data (x-axis) and correlated (Pearson’s correlations) them with the original posterior parameters estimates (y-axis), which is shown in plots a-f. The estimated parameters could be recovered well, however, the third level evolution rate (ωcard) could not be recoverd well. (TIF) [file pcbi.1008162.s013.tif]

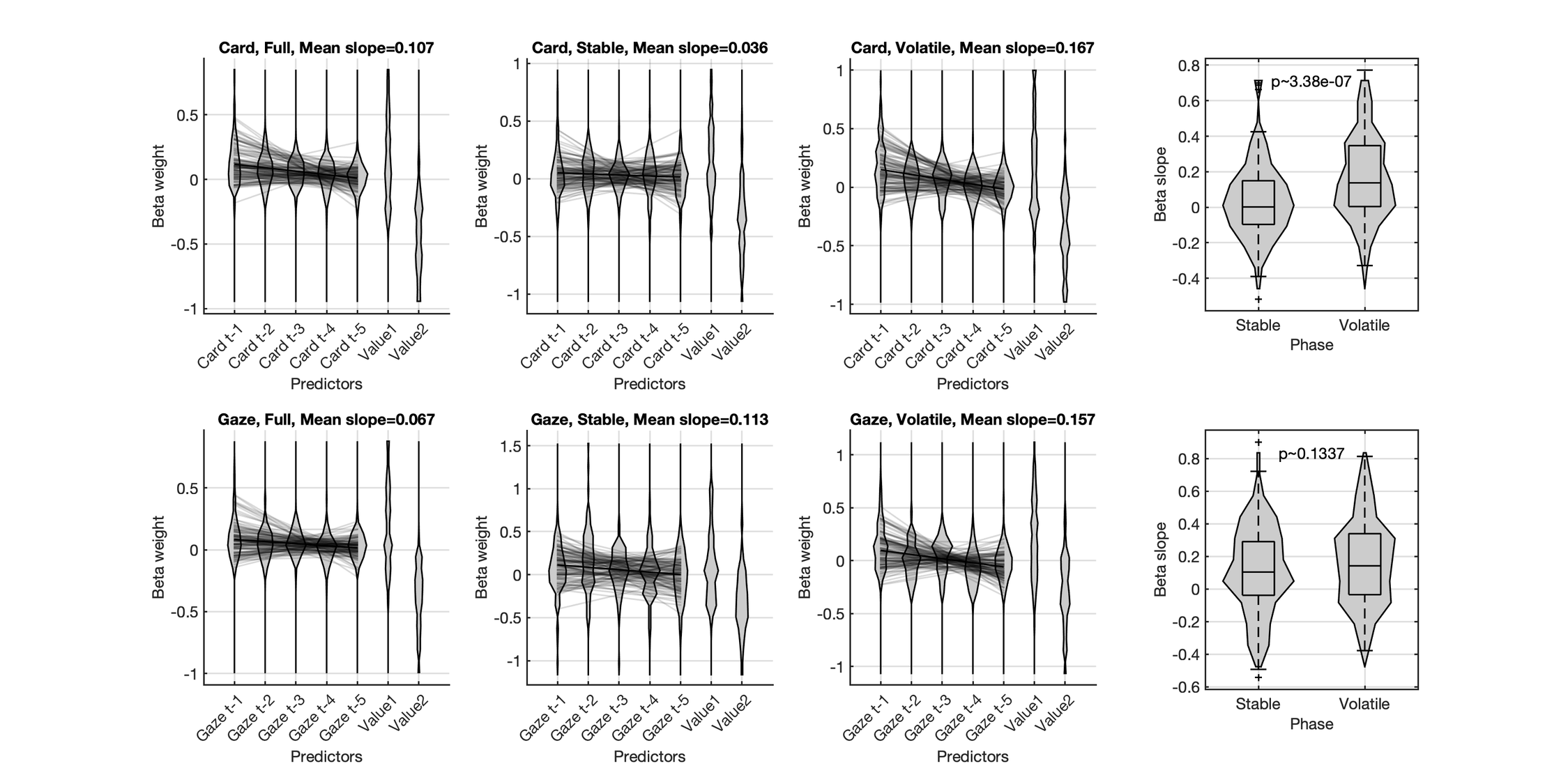

Supplement: S5 Fig — Top: Regression model for choices in card space (1 = taking blue, 0 = taking green) with predictors of card accuracy (1 = blue correct, 0 = green correct) for the past 5 trials, and reward values (reward value if blue taken, reward value if green taken) for the whole task (left), stable (middle) and volatile (right) phase. The slope between predictors were calculated as a model-agnostic readout of the ‘learning rate’, indiciating the degree to which more recent information is weighted. A t-test was applied to compare the difference between these slopes during stable and volatile phases of the task. Slopes are increasing during volatile compared to stable phases. Below: Regression model for choices in gaze space (1 = taking advice, 0 = not taking advice) with predictors of gaze accuracy (1 = gaze correct, 0 = gaze incorrect) for the past 5 trials, and card reward values (reward value if advice taken) for the whole task (left), stable (middle) and volatile (right) phase. (TIF) [file pcbi.1008162.s014.tif]
