# Supplementary material for: Aberrant computational mechanisms of social learning and decision-making in schizophrenia and borderline personality disorder
Source: PLoS Comput Biol. 2020 Sep 30;16(9):e1008162. doi: 10.1371/journal.pcbi.1008162 (PMC7588082; doi:10.1371/journal.pcbi.1008162)
Supplement: Attachment — Submitted filename: ResponsetoReviewers.docx [file pcbi.1008162.s015.docx]

**Manuscript title:** Aberrant computational mechanisms of social learning and decision-making in schizophrenia and borderline personality disorder

We are grateful to the reviewers for their insightful and constructive comments, which have helped us to further improve the quality of our manuscript. Please find a detailed point-by-point reply below. The paragraphs in normal blue font contain our answers while those in **bold blue font** contain the changes that were made to the manuscript.

After addressing all issues raised by the reviewers and making changes accordingly, we hope that the manuscript is now suitable for publication.

**Comments to the Authors:
Please note here if the review is uploaded as an attachment.**

Reviewer #1: In this paper, Henco et al. evaluate alterations in hierarchical evidence in social and non-social contexts across a transdiagnostic sample comprising individuals with schizophrenia, major depressive disorder, borderline personality disorder and healthy controls. They use a well-designed advice taking paradigm that manipulates of volatility and model the data using a variant of the 3-level hierarchical Gaussian filter, a widely used approach developed by this group. The experimental design and computational modeling is rigorous and sound and the transdiagnostic evaluation is a relevant and novel aspect of this work.

While this work has some relevant limitations, the strengths seem to outweigh them. I am generally enthusiastic about this work and only have relatively minor comments that I would like the authors to address.

1. One potential concern has to do with low-level confounds related to poor accuracy or use of heuristics. On Fig 3a, it seems like response accuracy is around chance level in some subjects. Can the authors provide evidence that these subjects understand task instructions and engage with the task as intended? Any basic manipulation checks would be useful. Furthermore, can the authors show that the group differences in fitted model parameters are not driven by low-level issues such as low overall poor accuracy or use of heuristics? Looking for strategies such as win-stay/lose-shift and the percentage of participants using them in each sample would also be advisable. Perhaps a within-subject model comparison could help exclude subjects for whom there is no evidence to support they are learning during the task.

Thank you for the positive assessment of the manuscript and the suggestions to perform additional analyses regarding decision-making heuristics.

Since the behavioral read-out refers to % correct (i.e., % rewarded) choices, we decided additionally to look at the % of correct choices based on the ground truth reward probability of both cues (see new Fig 2 and new behavioral results, line 452). Two things should be noted here. First, since there are two aspects to each decision, a social and a non-social one, each with their own ground-truth probability of being rewarded, there are trials where these two aspects conflict. On such trials, any decision will be classified as bad in one of the two aspects. This necessarily depresses measures of decision quality such as the one reported in Fig 2. Second, for the same reason, simply because a given subject performs poorly in one aspect of the task does not necessarily mean that this subject did not understand the task. They may simply be paying too much attention to the other aspect of it. Our new model-agnostic performance analysis by phase and by cue type shows that our experimental manipulations worked insofar as they had a different effect on different participant groups. Furthermore, this new analysis enabled to show that in particular BPD patients focus their learning on the social domain at the expense the non-social even when this is detrimental to their overall performance.

On line 403 of the methods section we now write:

**“In order to understand task performance in a domain-specific way, we additionally calculated response accuracy based on the ground truth reward probability (high probability choices) of both cues (non-social and social) and both phases (stable and volatile). The proportion of high probability choices was subjected to a mixed ANOVA with Cue Type (Non-Social vs. Social) and Phase (Stable vs. Volatile) as within-subject factors and Group (HC vs. MDD vs. SCZ vs. BPD) and schedule (congruent first vs. incongruent first) as between-subject factors.”**

On line 452 of the results section we now write:

**“There was a significant difference between the groups in the overall proportion of trials where the better option was chosen, i.e. proportion of correct choices based on the ground truth reward probability of both cues (*F*(3,108)=4.945, *p*=0.003,** $\boldsymbol{\eta}^{\boldsymbol{2}}$**= 0.116, Fig 2 and Table S6). Post-hoc comparisons showed that both SCZ and BPD patients performed significantly worse compared to HC (SCZ–HC *t*=3.373, *p*_bonf_=0.006, *d*=0.313, BPD–HC *t*=3.227, *p*_bonf_=0.01, *d*=0.3) across domains (i.e., cue types) (Fig. 2 and Table S6).Overall, response accuracy did not significantly differ between cue types (*F*(1,108)=2.577,  *p*=0.111,** $\boldsymbol{\eta}^{\boldsymbol{2}}$**= 0.019), but there was a significant Cue Type** $\boldsymbol{\times}$**Group interaction *F*(3,108)=4.820, *p*=0.003,** $\boldsymbol{\eta}^{\boldsymbol{2}}$**= 0.108), with HC (Post-hoc: t(30)=-2.157, p_bonf_=0.039, d=-0.387) and MDD patients (Post-hoc: t(27)=-2.181, p_bonf_=0.038, d=-0.412) showing higher response accuracy with regard to the non-social cue, whereas BPD patients showed the opposite pattern (Post-hoc: t(27)=2.058, p_bonf_=0.049, d=0.389). “**

In addition, we now include a win-stay-lose-shift (WSLS) model in our analysis, which assumes that participants repeat the actions that lead to reward and switch the strategy immediately after loss. In addition, we added a model which assumes random responding throughout the task. We did this by implementing the models suggested by Wilson & Collins (2019) in the HGF toolbox.

On line 351 we now write:

**“We additionally included two non-learning models, which assume that participants repeat the actions that lead to reward and switch the strategy immediately after loss (Win-Stay-Lose-Shift) and a model that assumes random responding throughout the task (random-responding). These models were adapted from Wilson & Collins (2019) and implemented in the HGF toolbox to calculate the log model evidence.”**

As you suggested, we performed within-subjects model comparison, identifying the best model for each subject (highest log model evidence), which can be seen in table S3. We ran the analyses excluding *all* subjects (16 of them) for which the random model showed the highest model evidence and show these results in Table S7-S9. This reduced sample showed the same result pattern as the full sample. Therefore, we decided to use the full sample for the analysis presented in the main text. The reduced sample results can be found in the supplementary material.

On line 364 we now write:

**“We additionally performed within-participant model comparisons to identify the model with the highest LME for each participant (cf. Table S4). This turned up 16 subjects where random responding had a higher model evidence than the overall winning model. Excluding these participants did not change the pattern of results (cf. Table S7-S9).”**

2. One important aspect of this study is the transdiagnostic evaluation of social learning impairments in relation to the ACIPS measure of social anhedonia, which the authors report as a negative finding. This result should be further emphasized in the discussion for balance. Previous work (e.g., Gillan et al.) suggests that transdiagnostic measures are more related to computational phenotypes than DSM categories, but this work seems to suggest otherwise. While this is a nuanced point, the authors should discuss the implications of this negative result along the lines of relevant previous work.

Thank you for this important remark. We have added a paragraph which expands on this to the discussion (line 694):

**“In addition to looking for learning and decision-making differences between the different diagnostic groups that were defined by traditional ICD-10 criteria, we adopted a transdiagnostic perspective to investigate the relation between computational mechanisms of social learning and decision-making with ACIPS, a self-report measure of social anhedonia. Previous studies have adopted such a dimensional approach in the general population and found that patterns in aversive learning mapped onto distinct symptoms of depression, social anxiety, and compulsivity [51,61,62]. We, however, could not find any transdiagnostic association of social anhedonia with computational parameters of social learning and decision-making in our data. A possible explanation for this negative finding is that we used scores from a single questionnaire whereas previous studies applied factor analysis (e.g. [61]) on all items from several questionnaires. In addition, these studies investigated larger samples compared to our study (>400 vs. 116) and therefore had more statistical power to detect signficant effects.”**

3. The authors make an effort to explain the partially convergent findings in schizophrenia and borderline personality disorders in terms of their shared symptoms, a relevant point that enriches the discussion. Obviously, these disorders also have many aspects that are not shared. Can the authors conduct further analyses to support which specific shared symptom domains may be driving the convergent findings across these two disorders? Can these findings be instead due to other confounds that may be shared between these two disorders or the specific samples included here, for instance similar medication or lower IQ or differences in sociodemographic status?

In the present study we investigated the transdiagnostic domain of social anhedonia and how it maps on the mechanism of social inference, which we report as negative findings.

It is possible that the convergent findings in borderline personality disorder may relate to psychotic ideation, which can be a shared feature of both disorders. Unfortunately, we cannot test this with the available data since patients with BPD did not answer the Positive and Negative Syndrome Scale. However, an association of psychotic ideation and social weighting as estimated in our study might not be sufficiently informative as the underlying mechanisms could still differ: Whereas patients with schizophrenia could tend to overweight social information in order to avoid intended harm by others, patients with borderline could tend to overweight social information in order to avoid being abandoned by others.

To control for the possible confounds with medication level mentioned by you above, we calculated chlorpromazine equivalents(cf. added to Table S2) and ran an ANCOVA with dependent variable $\zeta$ and Chlorpromazine Equivalence Units and Years of School as covariates (as we unfortunately do not have data on IQ). Neither covariate had a significant effect on the results.

On line 587 we now write:

**“To ensure that the shared mechanism of social over-weighting in BPD and SCZ was not confounded with medication or education status (cf. Table S1,S2), we subjected** $\boldsymbol{\zeta}$ **to an ANCOVA with**$\boldsymbol{\zeta}$ **and Chlorpromazine Equivalence Units and Years of School as covariates. The effects were robust to this.(cf. Table S9 for results of full and reduced sample).”**

4. Related to the previous point, one aspect I would like the authors to emphasize further is the integration of the current results with their previous results in autism and paranoia in the general population.

Many thanks for this helpful suggestion: In line 658 of the discussion we now write:

**“A previous study from our group found that healthy participants scoring high on AQ showed a similar pattern to the present study’s BPD patients, in that they followed the gaze more than low-AQ participants during periods of low accuracy in volatile phases[20]. In that study, computational modeling showed that high AQ participants failed to use the social information to adapt the precision of their belief about the non-social cue. However, this was not found in any participant group in the present study.”**

Additionally, in line 683 we now write:

**“Hypermentalizing is also a possible explanation for the findings of [60], where similar modeling as in the present study showed that healthy participants at the high end of the paranoia spectrum used similar weighting of social information irrespective of whether incorrect advice was framed to be intentional or not, while low-paranoia participants reduced their social weighting when negative advice was cued to be intentional.”**

5. It is unclear why the authors decided to simulate the data for a subset of participants in multiple iterations. Can’t the authors simulate data using the fitted parameter for each subject and take average responses for several iterations in a given subject as the responses in that subject so as to avoid differences in sample size and statistical power between the real and the simulated data? This needs clarification.

Thank you very much for this remark, we agree that it is better to simulate data for each participant instead of a subset of participants. We have now simulated responses for all participants10 times, resulting in 1160 simulations and calculated the average response accuracy (% of high reward probability choices as for the real data) for the two cues and the two phases. For the posterior predictive validity analysis, we subjected these values to the same ANOVA as performed with the real behavioral data to check if behavioral differences could be recovered, which they could.

In the methods section, line 370, we now write:

**“To test the adequacy of the model, we simulated responses based on the estimated parameters from the winning model for each participant 10 times, resulting in 1160 simulations. As for the real data, we then calculated percentage of high reward probability choices for the two cues and the two phases, subjecting them to the same ANOVA that was performed with the real behavioral data. In this way, we checked whether the simulated responses produce the same group differences in response accuracy.”**

In the result section, page line 511 we now write:

**“Subjecting the simulated behavioral readout of % of high probability choices to the same ANOVA as performed with the real behavioral data, we found that the model was able to reproduce the group differences that were observed in the real behavioral data (cf. table S6 for comparison). The ANOVA of the simulated data showed that for response accuracy, as in the real data, there was a main effect of Group (*F*(3,108)=6.755, *p*=0.001,** $\boldsymbol{\eta}^{\boldsymbol{2}}$**=0.149) with post-hoc t tests revealing significantly lower response accuracy for SCZ and BPD patients compared to HC (SCZ–HC *t*=4.04, *p*_bonf_<0.001, *d*=0.375, BPD–HC *t*=3.675, *p*_bonf_=0.002, *d*=0.341) and a Group** $\boldsymbol{\times}$ **Cue Type interaction (*F*(3,108)=10.981, *p*<0.001,** $\boldsymbol{\eta}^{\boldsymbol{2}}$**=0.219) showing that HC and MDD patients showed higher response accuracy with regard to the non-social cue, whereas BPD patients showed the opposite pattern (cf. Table S6 and Figure S2 for all results).”**

6. I would like for the authors add a **parameter recovery analysis** supporting the identifiability of parameters in their winning model, which tends to be a concern with highly complex models such as the one described here.

For the analysis of parameter recovery, we used the responses from the same simulation described above (point 5) to again invert the model. For each subject, we calculated the average posteriors estimated from the simulated data and correlated (Pearson’s correlations) them with the original posterior parameters estimates.

In the methods section, line 375 we now write

**“In a second step, simulated responses were again used to invert the winning model to check whether model parameters could be recovered. For each subject, we calculated the average parameter values estimated from the simulated data and correlated (Pearson’s correlations) them with the values estimated from the real data, which served as input for the simulation.”**

The results can be found in Figure S4 and show that parameter recovery worked well. On line 522, we wrote:

**“Parameter recovery showed that all parameters could be recovered well with the exception of** $\boldsymbol{\omega}_{\boldsymbol{3}\boldsymbol{card}}$ **(cf. Figure S4).”**

Minorpoints:

7. In Fig. 3 it would be preferable to show scattered data points for each subject (at least for the stable versus volatile contrast estimates).

To avoid Figure 3 being too cluttered, the original manuscript included Figure S2 which shows the grouped individual data points showing precision weights for updating beliefs about social and non-social contingency and volatility. We updated this figure, showing the data from the new model that was suggested by Reviewer #3.

8. Typo on line 149: I believe they mean ‘afforded’ instead of ‘accorded’

Thank you, we have corrected this mistake (now line 147)

9. To clarify, I am assuming these are all new samples, or have any of them been previously reported on? If so, please indicate which subsamples or parts of samples have been included in previous manuscripts.

Thank you for the remark. This is the first time this sample has been reported.

Reviewer #2: This study aims at assessing probability learning and volatility in schizophrenia (SCZ), borderline personality disorder (BPD) and major depressive disorder (MDD) as well as learning from social and non social cues.
The authors test patients diagnosed with MDD (N=29), SCZ (N=31), and BPD (N=31), and healthy controls (N=34) performing a probabilistic reward learning task in which participants could learn from social (a cue given by the gaze of a person in a photo) and nonsocial information regarding probabilities of two cards (a blue card and a green card) to give a reward. The probabilities of the pairing between each card and rewards could be stable or volatile. The social cue could be congruent or incongruent with the correct answer.
The individual behaviour data was fit using a HGF model.
Participant also filled an AQ questionnaire and a questionnaire regarding social anhedonia.
The raw data showed that BPD and SCZ had worse performance than MDD and HC with patients with BPD following the social advice significantly more compared to patients with MDD during volatile phases of low accuracy.
The modelling suggests impaired learning from social and non-social information in BPD characterised by an exaggerated sensitivity to changes in environmental volatility. Compared to controls, patients with BPD and SCZ showed an over-reliance on their beliefs about the predictive value of social relative to non-social information during decision-making.

The topic is very timely and interesting. The paper is clear and well written (though fairly technical due to being centred on the use of the HGF model). I think using this sort of task and modelling with patients having different types of ICD10 diagnostics and looking at commonalities or differences is very interesting and what needs to be done to progress in the field.

However, I have 2 major concerns about the study:

1) As the authors point out in Discussion, although the authors want to assess “social learning” (as per the title of the article), they didn’t use a control condition to assess whether the differences observed with the “social cue” had anything to do with the “social” nature of the cue (i.e. that it is presented as an eye-gaze), or whether it is more related to having to integrate two types of cues, that is whether they are looking at a difference in cue integration rather than in social learning, where the two cues might also rely on working memory in different ways. They could have used a control condition with an arrow for example instead of a gaze, and I don’t really understand why the authors didn’t do that. In my mind, it would have greatly enhanced the study and justified framing it as a study about “social learning”. In the absence of such a control condition, I find that all the arguments related to social learning to be weak and speculative.

Thank you for the positive feedback on our study and for adressing this limitation. While it is true that a non-social equivalent condition to the gaze cue could give additional weight to the ‘social’ interpretation of effects relating to that cue, we would like to emphasize that the gaze is an undeniably social cue while the card color is undeniably non-social. In this sense, we feel justified in interpreting contrasting results along the two dimensions in these terms. Having said this, we find that the between-group effects in *learning* are domain-general, i.e., they are not specific to social inference (line 665). However, we do find effects in decision-making that seem to be specific to the social domain. We agree with you that this should be further corroborated using additional control conditions in future studies. We acknowledge this on line 708, where we write:

**“We did not use a non-social cue (such as an arrow pointing to a card) as a control condition and therefore cannot fully rule out the possibility that the increased weighting of our social cue observed in BPD and SCZ reflects a more general rather than specifically social peculiarity in information processing. However, eye gaze is a very salient cue and in the paradigm, we aimed to accentuate the social quality of our cue by a clear period of eye contact with the participant before providing the cue.”**

2) I think because the study relies on the complex HGF model, it would require showing how good the model is at parameter and model recovery for this task/data (cf. Wilson and Collins, Ten simple rules for the computational modelling of behavioural data, eLife 2019). The model comparison with simpler models in particular the Rescorla-Wagner model (with fixed learning rate) doesn’t sound very fair: how does the RW model include the social cue?
It is important to justify the use of such complex models (rather than starting from them), and in my mind the way to do this is both to show that parameter and model recovery works, and that no simpler model can account for the data.

Thank you for this important remark. We fully agree with the suggested procedure of fitting simpler models to behaviour to test it against more complex models such as the HGF.

We have now extended our model space with the non-learning models suggested by Wilson and Collins (2019). These, together with the Rescorla-Wagner and Sutton-K1 model were compared against the Hierarchical Gaussian filter using Bayesian Model comparison.

We apologise if our description of how we implemented the Rescorla-Wagner and Sutton-K1 models was not clear enough. Both of these models were constructed in a way that they could compete fairly in the model comparison. The were both adjusted to model learning about both cards *and* social information (as was the HGF model) and were then combined within the response model just as the HGF. In effect, there were two RW and SK1 models working in parallel, one learning about color outcomes, the other about gaze cue accuracy, just as there were two HGFs doing the same.

In the previous version of our manuscript we wrote “We used the implementation of these models in the HGF toolbox and adjusted them in the same way as the HGF so that the model performs concurrent parallel learning about the social and non-social aspects of the task environment.”

To clarify this more, we now additionally write in line 245:

**“We used three different perceptual models in order to make inferences on the most likely mechanisms of learning in our paradigm. We used a Bayesian HGF as well as two non-Bayesian learning models, the Sutton K1 model [40] and a Rescorla Wagner model [41]. All three were implemented in the HGF toolbox, such that they perform parallel learning about the social (predictive value of gaze) and non-social (predictive value of card color) aspects of the task environment.**

**While the Rescorla Wagner learning model assumes fixed domain-specific learning rates for learning the social and non-social information, the Sutton K1 model assumes variable learning rates that are scaled by recent predicition errors. In contrast, the HGF takes into accout that beliefs have different degrees of uncertainty, scaling the learning rate dynamically as a function of uncertainty. In addition, the HGF assumes hierchical learning of different aspects of the environment. On the lowest level of the hierarchy, agents learn about concrete events (i.e. stimuli), whereas at higher levels of the hierarchy, agents learn about more abstract features of the environment, such as probabilistic associations between stimuli and how these change in time (i.e. volatility). Learning at every level is driven by a ratio between the precision of the input (from the level below) and the precision of prior beliefs.”**

With regard to model recovery, please refer to our answer to Question 6 of Reviewer 1 above.

Minor comments:

- Table 1 - explain how the reader is supposed to make sense of those numbers

On line 359 we now write:

**“This procedure estimates the expected posterior probabilities (EXP_P), i.e. the posterior probability of the prevalence of each model in the population, the exceedance probability (XP), i.e. the probability that a given model outperforms all others in the comparison, and the more conservative protected exceedance probability (PXP), which additionally considers the possibility that all models are equally good.”**

- Abstract and main text could be clearer in terms of what the behaviour shows, what questions it raises and what the modelling reveals in addition.

We have revised the abstract and it now reads as follows:

**“Psychiatric disorders are ubiquitously characterized by debilitating social impairments. These difficulties are thought to emerge from aberrant social inference. In order to elucidate the underlying computational mechanisms, patients diagnosed with major depressive disorder (N=29), schizophrenia (N=31), and borderline personality disorder (N=31) as well as healthy controls (N=34) performed a probabilistic reward learning task in which participants could learn from social and nonsocial information. Patients with schizophrenia and borderline personality disorder performed more poorly on the task than healthy controls and patients with major depressive disorder. Broken down by domain, borderline personality disorder patients performed better in the social compared to the non-social domain. In contrast, controls and MDD patients showed the opposite pattern and SCZ patients showed no difference between domains. In effect, borderline personality disorder patients gave up a possible overall performance advantage by concentrating their learning in the social at the expense of the non-social domain. We used computational modeling to assess learning and decision-making parameters estimated for each participant from their behavior. This enabled additional insights into the underlying learning and decision-making mechanisms. Patients with borderline personality disorder showed slower learning from social and non-social information and an exaggerated sensitivity to changes in environmental volatility, both in the non-social and the social domain, but more so in the latter. Regarding decision-making the modeling revealed that compared to controls and major depression patients, patients with borderline personality disorder and schizophrenia showed a stronger reliance on social relative to non-social information when making choices. Depressed patients did not differ significantly from controls in this respect. Overall, our results are consistent with the notion of a general interpersonal hypersensitivity in borderline personality disorder and schizophrenia based on a shared computational mechanism characterized by an over-reliance on beliefs about others in making decisions and by an exaggerated need to make sense of others during learning specifically in borderline personality disorder.”**

We have revised parts of the discussion with regard to the behavioral findings. In line 628 we now write:

**“In addition, patients with BPD showed greater response accuracy in the social compared to the non-social domain during the stable phase, while HC and MDD patients showed the opposite pattern. This is particularly remarkable in light of their overall poorer performance. In effect, BPD patients gave up a possible reward advantage by concentrating their learning efforts disproportionately in the social domain. In addition, we found a tendency in BPD patients to follow the gaze more during volatile phases of low accuracy compared to MDD patients, which however did not reach statistical significance (Fig. 3).**

**These findings raise the question which mechanisms underlie these patterns of behavior. In particular, they call for an investigation into the learning and decision-making mechanisms which give rise to them. Here, computational modeling enabled insights into how beliefs are updated and how these beliefs are translated into decisions: With regard to learning, we found that BPD patients showed increased precision weighting of prediction errors when learning about volatility in both non-social and social information and a tendency for even higher precision weights when learning about social compared to non-social volatility. While volatility learning rates** $\boldsymbol{(}\boldsymbol{\psi}_{\boldsymbol{3}}\boldsymbol{)}$ **were increased in BPD compared to HC, contingency learning rates (**$\boldsymbol{\psi}_{\boldsymbol{2}}$**) were reduced compared to HC both in the social and non-social domain.”**

Reviewer #3: The present study applied a probabilistic reinforcement learning task to three different groups of patients, major depressive disorder (MDD), schizophrenia (SZ), and borderline personality disorder (BPD), and healthy controls. The task allowed directly learning from the outcome of choices and about the reliability of a social cue (the gaze of a face presented centrally between the two choice options). Hierarchical Gaussian filter, a variant of Bayesian inference was applied to the behavioural data to extract different learning parameters. The main finding is that BPD show diminished learning from either source of information (outcomes and gaze information), whilst at the same displaying aberrantly high learning about the volatility of the environment (both the outcome and gaze, which orthogonally varied). Furthermore, choices of both BPD and SZ were guided more strongly by gaze compared to outcome information relative to healthy controls.

Overall, this is a very interesting study and I really appreciate the attempt at linking different clinical diagnoses with underlying computational phenotypes. However, at present, I am not fully confident about the findings presented. Specifically:

Thank you for the positive feedback and for providing helpful comments.

1) The group differences are exclusively observed in the model-derived parameters, not in any of the behavioural parameters. It is therefore very important to be able to follow exactly on what the HGF is doing. At present, I find the description of the HGF not very intuitive to follow for readers not familiar with Bayesian inference. Thus, one would have to take the authors' word for it. Rewriting this paragraph in a way that makes it more amenable to non-modellers could provide a lot more clarity - and make the work of greater interest to a broader readership.

We have extended our behavioral analysis and now also analyze the proportion of trials where the better option (according to ground truth reward probability in either domain, social and non-social) was chosen in addition to the % of correct (rewarded) outcomes (cf. Fig 2 and new behavioral results, line 452). This analysis showed that BPD patients disproportionately follow the social-domain probability trajectory compared to HC. The relevant passages are quoted in our response to reviewer #1 (major point 1 above).

We now also include a more intuitive description of the HGF (quoted above in our response to reviewer #2, major point 2).

2) Relatedly, I would find it very assuring if the authors could present a behavioural readout that does not rely on high-level Bayesian modelling. One option that comes to mind is to use a regression-based approach. Entering the (blue) outcomes and the gaze direction, each for the past n trials, reward available on the current trial (and possibly the interaction of both) could be used as predictors. This could be run separately for the stationary and volatile environments. In my view, the slope of the (social and non-social) weights should reflect the social and non-social learning rates, and the difference between blocks would reflect their adjustments in response to changes in volatility.

Thank you for the excellent suggestion to implement an additional model-agnostic analysis. We have now implemented a regression model with the responses in ‘card’ and ‘gaze’ space and the respective outcomes for the past 5 trials as predictors in addition to the rewards available on the current cards. We ran these GLMs for the stable and volatile phases separately. As you indicated, slopes of the regression weights increase during volatile phases as participants place more weight on recent outcomes. While this is observable for both cues, the effect is only significant for the card cue. This is most likely due to the nature of our task instructions, which do not tell explicitly participants to learn about the social cue. Therefore, participants who do not consider social information will have flat slopes for the social, but not for the non-social cue. This is accounted for in our behavioral modeling, which estimates the relative weight participants give these two types of information. Please see Figure S5 for the results of this additional analysis.

On line 381 we now write:

**“Regression-based choice sequence analysis**

**We ran a regression based choice sequence analysis in order to investigate adaptation to environmental volatility in without needing to fit a learning model. According to the HGF, agents should increase their learning rate in more volatile environments, giving more weight to recent outcomes. In more stable environments, agents should adopt lower learning rates, affording less weight to recent outcomes in order to better filter out noise. To test this in a model-agnostic way, we implemented two general linear models (GLMs) with the responses *y* as outcome variables for the ‘card’ and ‘gaze’ space respectively (card: 1=blue taken; 0=green taken; gaze: 1=follow; 0=not follow). For each GLM, we included as predictors the outcomes of the past 5 trials (t-1:t-5: 1=blue correct; 0=green correct) and (t-1:t-5: 1=gaze correct; 0=gaze incorrect) as well as two predictors for the expected reward (1-9) for either card winning. We ran these GLMs for the stable and volatile phases separately. The slopes of the coefficient estimates of the past outcomes predictors were taken as a model-agnostic readout of the ‘learning rate’, indiciating the degree to which more recent information is weighted. A t-test was applied to compare the difference between these slopes during stable and volatile phases of the task.”**

On line 530 of the results section we write:

**“In the model-agnostic regression analysis, we observed increased slopes of beta weights over time delays in the volatile vs. stable phase (Fig S5) indicating higher weighting of most recent trials, which concurs with the increased learning rates in the volatile phase in the HGF. While this effect was observable for both cues, the difference in the model-agnostic analysis was significant only for the card cue, perhaps because this analysis does not consider individual variations in cue use.”**

3) It is currently hard to judge how well the best fitting model described participants' choices. Could you please provide some more intuitive measure, such as the **model's average probability** of choosing the option selected by the participant? Is the winning model the same in all patient groups/controls? In how many of the participants was it the best one?

We checked how well the estimated combined belief of gaze accuracy of every participant (Manuscript, Equation 6) mapped onto decisions to follow the gaze or not. The winning model’s average probability for the participant choosing the option they actually went on to choose was 0.62 across all participants. Broken down by participant, the median and interquartile range were 0.61 and 54.2- 68.8, respectively. This correlated positively with the Log Model evidence (rho=336, p<0.001). As partly already described in our response to reviewer #1 (major point #1), we additionally performed within-subjects model comparison to identify the model with the highest LME for each participant. The results are shown in Table S3. The winning model was best in 47 participants, Sutton K1 in 16, random choice in 16, Rescorla-Wagner in 14, win-stay-lose-shift in 11, HGF2 in 8, and HGF3 in 4, and HGF4 in none.

Beyond the results of model comparisons, a strong reason for us to choose the winning model was that it is the only model we could use to test our research question regarding precision imbalances when inferring on the probability *and* volatility of non-social and social hidden states cross-diagnostically.

4) Only two non-Bayesian models are presented, and these are not explained, the reader is referred to two references. While I know what an RW model is, I had not heard about the Sutton K1 model before, and even a simple RW model can come in many flavours. What was it that motivated the choice of these specific two non-Bayesian models? Why not others, e.g. some that also feature dynamic learning rates (e.g Pearce-Hall associability), or separate learning rates for social and non-social information? There are various other alternative models that could be compared here, and I am not sure why the authors restricted it to those two.

We are sorry for the brevity of our model choice explanation. We have now greatly expanded this (quoted above in our response to reviewer #2, major point #2. We have also expanded our model space (as explained above reviewer #1, major point #1). However, we think it important to note that we used these comparisons mostly as a sanity check since only models allowing for a readout of beliefs about volatility allowed us to address our hypotheses regarding group differences in this respect.

5) I am also missing details on the task. What was the range of possible outcomes? I am asking because (as described by prospect theory) humans typically do not weight reward amount linearly, tending to underweight higher amounts. Does it improve model fits to account for such subject-specific distortions?

We are sorry if this was unclear, and we thank you for your useful suggestion to check whether non-linear weighting of expected outcomes would improve our model fit. We augmented our response model to be a weighted average of the previous response model, where the combined belief of gaze accuracy was **linearly** weighted by the expected reward, with a corresponding **logarithmically** weighted model (cf. MS, Equation 11). All 6 learning models were combined with this new weighted-average response model and were fit to behavioural data. We compared the original, linearly weighted response model against the new model by means of random effects family-wise model comparison (Penny et al., 2010). The new model family (EXP_r=0.878, XP=1) outperformed the original model family with linear reward weighting (EXP_r=0.122, XP=0). All results we report are now based on this new response model. However, this does not lead to any qualitative change in the results.

On line 321 we now write:

**“We took account of possible subject-specific non-linear distortions in weighting the expected reward by using a weighted average (parameter** $\boldsymbol{\eta}$**) of linear and logarithmic weighting of expected reward.”**

We changed the Equation 11 accordingly:

$$\boldsymbol{prob}_{\boldsymbol{gaze}}\boldsymbol{=}\boldsymbol{p}\left( \boldsymbol{y}^{\left( \boldsymbol{t} \right)}\boldsymbol{=1} \right)\boldsymbol{=\eta}\boldsymbol{1}/\left( \boldsymbol{1+}\exp\left( \boldsymbol{-}\boldsymbol{\gamma}^{\left( \boldsymbol{t} \right)}\left( \boldsymbol{r}_{\boldsymbol{gaze}}^{\left( \boldsymbol{t} \right)}\boldsymbol{b}^{\left( \boldsymbol{t} \right)}\boldsymbol{-}\boldsymbol{r}_{\boldsymbol{notgaze}}^{\left( \boldsymbol{t} \right)}\left( \boldsymbol{1-}\boldsymbol{b}^{\left( \boldsymbol{t} \right)} \right) \right) \right) \right)\boldsymbol{+} \left( \boldsymbol{1-\eta} \right)\boldsymbol{1}/\left( \boldsymbol{1+}\exp\left( \boldsymbol{-}\boldsymbol{\gamma}^{\boldsymbol{(}\boldsymbol{t}\boldsymbol{)}}\left( \log\left( \boldsymbol{r}_{\boldsymbol{gaze}}^{\left( \boldsymbol{t} \right)} \right)\boldsymbol{b}^{\boldsymbol{(}\boldsymbol{t}\boldsymbol{)}}\boldsymbol{-}{\mathbf{log}\boldsymbol{(r}}_{\boldsymbol{notgaze}}^{\left( \boldsymbol{t} \right)}\boldsymbol{)}\left( \boldsymbol{1-}\boldsymbol{b}^{\boldsymbol{(}\boldsymbol{t}\boldsymbol{)}} \right) \right) \right) \right)$$

6) In the results, it says: "We simulated responses using the posterior mean parameter values of 60 randomly chosen participants from the best fitting model to demonstrate that this model was capable of reproducing the group differences that were observed in the real behavioral data..."
This seems arbitrary. Why only a subset (of more than 50% of all participants) rather than all participants? Furthermore, can the fitted parameters be recovered from the simulated data?

We agree with this comment. Please refer to our answer to Reviewer 1, point 5 and 6.

7) Overall performance (% correct) is impaired in BDP and SCZ, and overall task performance is quite low. In particular in the patient group, there appears to be a significant fraction of people that performs just around chance level (some slightly above, some even below). This leaves me wondering whether there was a substantial proportion of participants (patients) that failed to properly perform the task?

Thank you for this useful remark. Please refer to our answer to Reviewer 1, point 1.


Minor:
1) How did you define "% correct"? Does this refer to choices of the option with higher probability or with higher expected value (probability x points)?

Thank you for this question. We defined % correct with respect to rewarded trials. Please also refer to our response to your major point #1 and reviewer #1 (major point #1) for the new behavioral analysis.

2) Figure 2: please indicate in the legend what values are shown. It looks like medians in 2A+B, but mean ± SEM in 2C? Please clarifiy.

We are sorry about this imprecision on our part. We have now added the following to the caption of the new figure Fig.2 and Fig.3.

On line 480 and 487 we now write:

**Means are plotted with boxes marking 95% confidence intervals and vertical lines showing standard deviations.**

3) Parts of the abstract appear to be redundant, unless I'm mistaken, lines 72-76 merely repeat the findings already stated above?

Thanks for making us aware of these redundancies. Please refer to our answer to reviewer #2, minor point #2 for the new version of the abstract.

Additional changes to manuscript by us:

We have added a short discussion of our negative findings regarding MDD. We now write on line 670:

**“The same applies to MDD patients, where one possible explanation for this negative finding is the lack of punishment for incorrect choices in our task since recent findings converge on impaired aversive learning in depression (e.g. [51,52]).”**

We have added a short paragraph discussing the findings of social over-weighting in SCZ and BPD in light of a recent fMRI study from our group in HC. We now write on line 687:

**“Furthermore, a study of healthy participants by our group [30] found that stronger weighting of social over non-social predictions during decision-making was associated with increased activity in the putamen and anterior insula. In future studies, it will be interesting to investigate the involvement of these regions in excessive social-weighting and hypermentalizing in BPD and SCZ. Also, a direct comparison of patients with BPD and autism could help to unveil shared mechanisms of aberrant social inference.”**
